# Combined genetic polymorphisms and environmental factors in the etiology of a chronic TMJD murine model

**DOI:** 10.1186/1744-8069-10-S1-O18

**Published:** 2014-12-15

**Authors:** Karin N Westlund, Sabrina L McIlwrath, Helieh S Oz

**Affiliations:** 1 Department of Physiology, University of Kentucky, Lexington, KY 40536-0298, USA

## Background

TNFR1 and TNFR2 are receptors for the pro-inflammatory chemokine TNFα. Inflammation of the temporomandibular joint (TMJ) and hypersensitivity were induced in mice lacking both TNFα receptors (TNFR1/R2 -/-). After recovery from this initial priming TMJ insult lasting less than a week, a gastrointestinal (GI) chemical irritation is administered 3 weeks later. This “double-hit” initiates a recrudescence of inflammation and hypersensitivity persisting at least 18 weeks.

## Materials and methods

TNFR1/2 -/- and wildtype (WT) mice were given a unilateral TMJ injection of complete Freud’s adjuvant (CFA, 5-10 mL) to induce hypersensitivity of the overlying facial area. Mechanical hypersensitivity threshold was determined with graded nylon von Frey monofilaments. Heat hypersensitivity was determined by latency to response with the hotplate test (50°C). Three weeks later, mustard oil (0.5% in peanut oil; 50 μl) was infused into the colon. The prolonged inflammation and hypersensitivity that re-developed only in the TNFR1/2-/- mice allowed testing of several different compounds for their efficacy to reduce chronic inflammation-induced mechanical and heat hypersensitivity. Proteomic analysis of serum was performed to study cytokine level differences in weeks 2 and 10.

## Results

While mechanical thresholds and heat response latencies in WT mice returned to baseline after both insults, the TNFR1/R2 -/- mice developed chronic mechanical and heat hypersensitivity which persisted at least 18 weeks. NMDA receptor antagonist MK801, P2X7 inhibitor A438079, reactive oxygen species scavenger phenyl-N-t-butyl nitrone (PBN), and human TNFα neutralizing antibody Etanercept were tested to determine efficacy for reduction of the hypersensitivity. Both mechanical and heat hypersensitivity were attenuated by the test drugs with varying efficacies. A TRPV1 antagonist, capsazepine, did not alter established heat hypersensitivity. At weeks 2 and 18, the TNFR1/R2 -/- and WT mice had very different cytokine profiles. At 2 weeks when the initial inflammation-induced hypersensitivity had resolved, serum levels of TNFα, RANTES, and MIG were twice as high in TNFR1/R2 -/- while CXCL11 was decreased compared to WT mice. After 18 weeks when hypersensitivity is chronic, G-CSF, IFNγ and TNFα serum levels in TNFR1/R2 -/- animals were significantly increased relative to the controls IL-16 and CXCL9 were decreased compared to WT samples.

## Conclusions

Utilizing a “double hit” inflammatory model we demonstrated that TMJ inflammation can be “re-ignited” by a minor GI insult to become a chronic condition in mice with TNF receptor deficits. High levels of TNFα were accompanied by different inflammatory mediators after the acute inflammation compared to those present chronically with the TMJD recrudescence.

## Disclosures

The authors have no financial conflicts of interest.

